# Attriters and Bilinguals: What’s in a Name?

**DOI:** 10.3389/fpsyg.2021.558228

**Published:** 2021-07-15

**Authors:** Federico Gallo, Keerthi Ramanujan, Yury Shtyrov, Andriy Myachykov

**Affiliations:** ^1^Centre for Cognition and Decision Making, Institute for Cognitive Neuroscience, HSE University, Moscow, Russia; ^2^Centre for Neurolinguistics and Psycholinguistics, Vita-Salute San Raffaele University, Milan, Italy; ^3^Center of Functionally Integrative Neuroscience, Aarhus University, Aarhus, Denmark; ^4^Department of Psychology, Northumbria University, Newcastle upon Tyne, United Kingdom

**Keywords:** bilingualism, L1 attrition, bilingual experience, L2 immersion, sociocultural changes

## Abstract

The use of language as a universal tool for communication and interaction is the backbone of human society. General sociocultural milieu and specific contextual factors can strongly influence various aspects of linguistic experience, including language acquisition and use and the respective internal neurolinguistic processes. This is particularly relevant in the case of bilingualism, which encompasses a diverse set of linguistic experiences, greatly influenced by societal, cultural, educational, and personal factors. In this perspective piece, we focus on a specific type of linguistic experience: non-pathological first-language (L1) attrition—a phenomenon that is strongly tied to immersion in non-L1 environments. We present our view on what may be the essence of L1 attrition and suggest ways of examining it as a type of bilingual experience, in particular with relation to its neurocognitive bases.

## Introduction

Globalization and mobility are increasingly establishing themselves as defining features of current world. Reports of the United Nations highlight a steady yearly growth in migration; in the last 20 years, the phenomenon doubled in magnitude, with the number of migrants reaching the figure of 260 million in 2017 (United Nations, 2018). As a result of such mobility, alongside changes in educational requirements, and internationalization of the job market, more than half of the world population is currently estimated to be bilingual (e.g., Grosjean, 2010). For instance, bilingual citizens constitute 21.5% of the grand total in the United States population (American Community Survey, 2015), 17.5% in Canada (Canada Census Program, 2011), and 54% in the European Union, where over 90% of the bilingual population reached peaks in some countries (Eurobarometer Report “Europeans and their languages”, 2012). A unique challenge for the bilingual mind is the simultaneous storage and management of two or more linguistic codes, which have been shown to be in a constant interaction with each other. In fact, a considerable amount of literature has investigated and demonstrated the influence of the first-language (L1) on the second one (L2; e.g., Dijkstra and Van Heuven, 2002). The equally important and plausible effects of the L2 on the native language have received much less attention. Here, we focused on one specific type of language experience that is closely associated with the latter—*L1 attrition*—the non-pathological, gradual decrease of native language performance that takes place alongside with, and even without (Baladzhaeva and Laufer, 2018), increase in L2 proficiency (Köpke and Schmid, 2004). Research on L1 attrition emerged in the early 1980s, and at present, almost 40 years later, still occupies a relative niche in the field of bilingualism. Nonetheless, a great progress has been made in the last two decades, due to a remarkable effort from attrition researchers to affine definitions and develop tools to investigate the phenomenon and its underlying mechanisms.

In this short opinion piece, after briefly defining the concept of L1 attrition and reviewing its putative underlying mechanisms, we advocated for the continuation and reinforcement of a trend put forward by attrition researchers in the last 15 years, unifying the characters of the bilingual and the attriter within the shared theoretical framework of crosslinguistic interaction. Our goal is to appeal to “traditional bilingualism” researchers to firmly establish the attrition phenomenon in empirical investigations as well as in theoretical platforms, as accounting for “the other side of the coin” can help to shed light on the neural and cognitive phenomena of the bilingual mind.

## What Is L1 Attrition?

Among the many existing characterizations of this phenomenon, described by Köpke (2004) as a “terminological jungle,” L1 attrition has been broadly defined as “any of the phenomena that arise in the native language of a sequential bilingual as the consequence of the co-activation of language, crosslinguistic transfer or disuse” (Schmid and Köpke, 2017a, p. 637)^1^. Guided by this view of L1 attrition, there has been an extensive characterization and a detailed analysis of how the linguistic behaviors of bilingual attriters, particularly those who are captured in productive language and “offline” tasks, contrast with those of monolingual speakers. Examples of such studies include research in L1 accent/phonology attrition (Bergmann et al., 2016; de Leeuw et al., 2018), analyses of morphosyntactic reconfigurations (Karayayla and Schmid, 2019), and the changes in L1 fluency and complexity induced by L2 exposure (Schmid and Jarvis, 2014; Bergmann et al., 2015a). From this viewpoint, linguistic behavioral deviations from monolingual standards are considered as indicative of L1 attrition. While there is extensive evidence of L1 attrition arising from L2 interference (see, Köpke and Schmid, 2004; Schmid and Köpke, 2017a), some studies also report L1 attrition occurring in the absence of an L2. For example, Laufer and Baladzhaeva (2015) and Baladzhaeva and Laufer (2018) investigated lexical, grammatical, and morphosyntactic L1 attrition in a sample of Russian immigrants in Israel with no knowledge of Hebrew as L2, comparing them with immigrant Russian/Hebrew bilinguals and Russian monolinguals still residing in Russia. Their results suggested that L1 linguistic behavior is susceptible to change even without the explicit knowledge of an intervening linguistic system. While L2 interference might still have made a contribution to L1 attrition in the latter case *via* a possible passive exposure to Hebrew spoken within the bilingual Russian immigrant population, the lack of explicit knowledge of L2 in the attriting population indicates a potentially intricate and complex nature of the attrition phenomenon.

This intricate phenomenological nature begs the following question: *What is L1 attrition*? To answer this question, we (1) raised two more questions of why and how it occurs, reviewing recent findings and (2) endorsed a research strategy that builds upon and might contribute to recent developments aimed at unifying attrition and bilingual research fields. We acknowledged that, in posing these questions, there is an unavoidable circularity problem—each question rests on the assumption that L1 attrition is already defined despite this being the very thing that one hopes to achieve (we discussed a possible solution in section “Who is the L1 attriter—a bilingual by another name?”). Nevertheless, the answers to these interconnected questions may contribute toward qualifying the phenomenon of L1 attrition, and equally importantly they may help us understand how L1 attrition relates (and, essentially, belongs) to the general phenomenon of bilingualism.

### Question 1: *Why* Does Attrition Occur?

First, there appears to be a certain selectivity of L1 attrition effects, i.e., there is considerable interindividual variation both in the severity of its “symptoms” and in the types of linguistic structures and systems it affects (Schmid, 2014). Thus, an alternative question, and perhaps one that is more specific would be, “*When* does attrition occur?”—i.e., under what specific conditions are L1 attrition effects most likely to appear?

It may be informative to begin by considering a situation in which L1 attrition disappears. A return (even if temporary) to the L1-dominant/native environment induces a rapid reversal of L1 attrition effects (e.g., Chamorro et al., 2016b; Gargiulo and van de Weijer, 2018; Köpke and Genevska-Hanke, 2018). On the one hand, this phenomenon suggests a potential role by the relative *quantity* and *quality* of contact with L1 as reimmersion in the L1-dominant environment brings better, more frequent opportunities to use L1 of an individual. On the other hand, it reinforces the role of L2 in inducing and driving changes in L1 as contact with L2 is naturally reduced upon a return to the L1 environment. In fact, experimental evidence has pointed at quantity (e.g., de Bot et al., 1991; Isurin, 2007; Opitz, 2013; Bergmann et al., 2016; Chamorro et al., 2016b; Kasparian et al., 2017; Schmid and Yilmaz, 2018; Karayayla and Schmid, 2019) and quality (e.g., Schmid, 2007; de Leeuw et al., 2010; Schmid and Dusseldorp, 2010; de Leeuw et al., 2012; Yilmaz and Schmid, 2012) of L1 exposure and at L2 interference (e.g., Ben Rafael, 2001; Dussias, 2004; Hutz, 2004; Ventureyra et al., 2004; Ribbert and Kuiken, 2010; Schmid and Jarvis, 2014; Chamorro et al., 2016a; de Leeuw et al., 2018) as factors contributing to the presence and the severity of L1 attrition.

Besides these main causal factors, however, research has also highlighted a number of other key variables that can shape the L1 attrition experience of an individual, including the length of residence in an L2-dominant country, age of migration, attitude toward L1 and L2, communal/social identity and affiliation, extent of social integration, socioeconomic status, and age. Since an extensive and detailed review of experimental findings goes beyond the scope of this opinion, we redirected the reader to a recent work by Schmid et al. (2019) and references therein for a thorough account of existing evidence. It is important, however, to note that these factors are not exclusive to L1 attrition, but that they also contribute to variation in the bilingual experience *per se*. Nevertheless, taking together both the selectivity and reversibility of L1 attrition effects, it seems that whatever L1 attrition is, it does not appear to involve the actual erasure of tacit linguistic knowledge or representations. This brings us to the next question.

### Question 2: *How* Does Attrition Occur?

Considering what we know thus far, one might ask: Is L1 attrition a stand-alone phenomenon, resulting (perhaps temporarily) from a qualitative and quantitative reduction in L1 use (with or without L2 exposure) or does it emerge due to the words, categories, and rules of L2 interfering with those of L1? Exploring what L1 attrition looks like in the mind and the brain of those experiencing it may help us find possible answers to this question.

Changes in observable linguistic behavior (measured using free speech and “offline” grammaticality judgments) and productive language suggest a possible change in the underlying neuro-psycho-linguistic processes that support such behaviors. The detailed analyses and descriptions of L1 attrition tell us how it may manifest at the behavioral end point (see text footnote 1 for examples of such studies). However, behavioral investigations alone can only hint at *neurocognitive mechanisms* underlying L1 attrition. Electrophysiological and neuroimaging techniques permit simultaneous investigations of both ends of the brain–behavior loop. Thus, examining “L1 attrition” from the point of view of how L1 language processing mechanisms have (or have not) changed might potentially bridge the existing brain–behavior knowledge gap in these studies (see Kasparian and Steinhauer, 2017b; Smith, 2019, for a similar view). In fact, such studies have emerged in recent years (see Schmid et al., 2019, for a comprehensive collection), and they reveal very interesting findings. For instance, using electrophysiological measures (ERPs—event-related potentials), Kasparian et al. (2017) and Kasparian and Steinhauer (2016, 2017a) have shown that certain forms of L1 morphosyntactic processing in L1 attriters are indeed different from that of non-attriting monolinguals.

One advantage of studying L1 attrition using neurophysiological and neuroimaging techniques is that it can help uncover neurocognitive features possibly characterizing attrition even in instances where it does not manifest in external linguistic behaviors. For example, some studies show that attriting bilinguals perform no differently than the non-attriting controls in offline behavioral sentence judgments, while the ERP data show specific group differences: for instance, where German monolinguals exhibit a posterior P600 effect to verb form violations, attriters show a biphasic N400-P600 pattern (Bergmann et al., 2015a). Similarly, Italian attriters display a more temporally distributed late P600 in response to anomalous sentences, while non-attriting peers exhibit only small P600 effects (Kasparian and Steinhauer, 2016). Taken together, these findings suggest that despite offline linguistic performance parity, underlying neurocognitive computations in L1 attriters proceed somewhat differently. In fact, investigating L1 attrition by capturing internal neurolinguistic and neurocognitive processes will be particularly useful in understanding the various attrition experiences (e.g., Laufer and Baladzhaeva, 2015), going beyond their apparent external presentations.

## Who Is the L1 Attriter—A Bilingual by Another Name?

The two questions posited above motivate a very important third question: *Who are L1 attriters?* Are they defined by (1) external circumstances, e.g. immigration to an L2 environment, (2) their apparent linguistic behaviors, e.g., changes/reconfigurations in L1 production or offline performance deviations involving L1 morphosyntax, or (3) a specific set of internal neurocognitive states and processes? Equally important question is that is attrition really that separable or distinct from bilingualism *per se*?

It is recalled that Schmid and Köpke (2017a) regarded L1 attrition to be the “effect of the second language on the first” and, by relabeling the crosslinguistic influence of L2 on L1 as L1 attrition, they highlighted that “all bilinguals are attriters” (Schmid and Köpke, 2017b). Here, we shifted the focus to the complementary argument that “all attriters are first and foremost *bilinguals*” to highlight the point that L2 → L1 effects are non-separable from bilingualism and that “attrition” is indeed a sub-phenomenon of bilingualism. In fact, the key point of L1 attrition research is that not only the L1 influences the L2 but the L2 also affects the L1. A different terminological choice must not create barriers between overlapping research fields—this dynamic interaction of two languages has long been regarded as one of the most defining features of bilingualism (Kroll et al., 2012, 2015). Crosslinguistic interplay (L2 ⇄ L1), something that monolinguals obviously lack, is arguably the greatest influence behind the distinctive organization and functioning of the bilingual mind and brain (Hernandez et al., 2005, 2019a; Hernandez, 2013; Li et al., 2014; Bialystok, 2017; Hayakawa and Marian, 2019). In fact, there is currently a substantial amount of empirical evidence demonstrating the specific ways in which L1 and L2 of the bilinguals are affected by one another (Kroll et al., 2012; Coderre, 2015). While the effect of a dominant L1 on a later acquired, relatively less proficient L2 might be expected and considered natural, there is also substantial evidence for an effect of the L2 on the L1. Various types of bilinguals, even in communicative contexts requiring exclusive use of their L1, exhibit certain behaviors due to unavoidable L2 influence that their monolingual peers do not—these include slower word production, decreased accuracy, lower semantic fluency, and increased tip-of-tongue states in their L1 (Bialystok et al., 2012; Costa and Sebastián-Gallés, 2014; Kroll et al., 2015). These are accepted as a natural outcome of housing two *interacting* language systems and are hardly ever labeled as instances of attrition. It is possible that the linguistic behaviors considered to amount to L1 attrition could be due to *changes in the L1–L2 dynamic*, changes in the extent to which the two languages influence one another, brought on by experiential changes including migration to different sociolinguistic environments, and not just purely due to the effects of an L2 on the L1, which persist regardless in *all* bilinguals. Similarly, Hernandez et al.(2019b, p. 260) suggested that the bilingual system is non-linear and dynamic (Hernandez et al., 2019a) and that attrition indicates its reconfiguration(s) and repurposing to suit new contexts. This is something that attrition research has been acknowledging for a long time (Schmid and Köpke, 2007). Thus, as also proposed by Schmid and Köpke (2007), the relevance of L1 attrition for the theories of bilingual development is reinforced by the fact that L1 attrition appears not to be intrinsically distinct from bilingualism, but rather a feature of the latter.

To determine whether or not L1 attrition is qualitatively distinct from bilingualism as such, one would need to determine features that are attributable to L1 attrition alone. However, in nearly all attrition studies thus far^2^, the L1 attriters are bilinguals, while the non-attriting controls are mostly monolinguals. Thus, the comparisons are not really between “attriters” and “non-attriters” but between those whose L1 surely receives some influence from the L2 and those whose L1 is free from it. Consequently, the findings of group-level linguistic and behavioral differences taken as an indication of L1 attrition can be seen as consequences of bilingualism and fall within the typical range of behaviors that bilinguals exhibit owing to their specific linguistic situation (e.g., see Bergmann et al., 2015b).

This interpretation is equally applicable to the existing ERP studies where the key ERP differences between attriters and monolingual controls might actually reflect a bilingualism artifact. For example, Bergmann et al. (2015a) acknowledged that the biphasic N400-P600 pattern in their bilingual attriters may reflect the relative linguistic difference between the L1 and L2 and is similar to findings reported by Sabourin and Stowe (2008), who examined how L1–L2 similarity alters language processing in (non-attriting) bilinguals. Similarly, Kasparian and Steinhauer (2016) noted that the larger P600 response that only their attriter group exhibited is possibly suggestive of increased conflict-monitoring and “re-checking” while processing anomalous lexico-semantic components in sentences. The fact that bilinguals, compared with monolinguals, rely on the increased extent of conflict monitoring while processing either language to control and manage crosslinguistic influence is empirically well established (Bialystok, 2017; Calabria et al., 2018). However, if the attriting samples in both studies were to be compared with a non-attriting bilingual group that has managed to maintain L1 use (with or without migration), it would tell us whether or not the specific P600 response (signaling increased conflict monitoring) or the biphasic responses (modulated by linguistic distance) are unique to the attriters. In this case, it could qualify as a specific L1-attriton marker; otherwise, if it is shared with bilingual controls, this would signify a bilingualism effect arising due to the presence of two interacting linguistic systems but not a distinct marker of attrition. To the best of our knowledge, no such studies have been done to date.

Thus, we supported the view that L1 attrition, characterized by a relative increase in frequency, quantity, quality, diversity of L2 use, and exposure and a possible concomitant decrease in all these aspects for the L1, be included within the spectrum of bilingual experiences. We further encouraged researchers to view attriters as a group within the *bilingual spectrum* and sought a better understanding of how distinct they may be, if at all. Further neurocognitive and behavioral comparisons between *attriting and non-attriting bilinguals* (e.g., Major, 2010; Schmid, 2014; Miller and Rothman, 2020) are needed to continue unveiling *what* L1 attrition really is and determine whether there are indeed linguistic behaviors and associated neurocognitive processes that are distinct and separable enough from the range of crosslinguistic bilingual effects to be characterized as L1 attrition. Findings from extant L1 attrition research also seem to reinforce the notion of “heterogeneous outcomes of heterogeneous bilingual experiences.” For example, increasing length of immersion, L2 exposure, and L2 proficiency has been shown to modulate L1 ERP responses among bilingual “L1 attriters” themselves (e.g., Kasparian and Steinhauer, 2017a; Miller and Rothman, 2020). This is in line with recent findings demonstrating that variations in the aforementioned bilingual experiential factors have a discernible impact on the linguistic neurobiology, neurocognition, and behavior of the bilinguals (e.g., Gullifer et al., 2018; DeLuca et al., 2019; Gallo et al., 2021; Sulpizio et al., 2020).

Finally, we addressed the circularity problem mentioned earlier: to study L1 attrition, we already needed to have an idea of *what* it is and where to find it. In order to break free from the risk of circularity, i.e., defining the population of interest in terms of the concept of interest, one needs to determine and adopt attrition-free criteria for identifying the population of interest. Based on the well-documented evidence of L1 attrition effects, we stated with reasonable confidence that bilinguals immersed in an L2-dominant sociocultural environment due to migration are clearly the population of interest here—they are most likely to exhibit behavioral and neurocognitive characteristics that might differ from their bilingual peers elsewhere, especially those in *L1*-dominant environments. Rather than immediately labeling the migrant bilinguals as L1 attriters, we could qualify and quantify their dual-language experience accompanying migration, acculturation, and immersion in an L2-dominant region as just another variant of the bilingual experience. Comparing this L1-attrition or disuse type of bilingual experience to other experiences on the bilingual spectrum, such as the L1-maintenance experience of non-migrant bilinguals in L1-dominant regions or of those who have managed to maintain active dual-language use despite migration, might generate enough empirical evidence that would allow us to deduce whether or not L1 attrition is wholly distinct and orthogonal from bilingualism.

## Conclusion

Attrition experience is highly typical for bilinguals who have migrated to non-L1 environments, which motivates understanding of the L1 attrition phenomenon as a distinct type of bilingual experience. It is therefore important to provide comparisons involving migrant bilingual populations with suitable bilingual controls (besides monolingual ones) on linguistic behaviors as well as on the underlying neurocognitive processes. Studying L1 attrition from the vantage point of bilingualism would not only contribute toward enriching and informing our current understanding of bilingualism itself, but it will also offer a richer perspective on how sociocultural factors shape linguistic behaviors and processes.

## Data Availability Statement

The original contributions presented in the study are included in the article/supplementary material, further inquiries can be directed to the corresponding author/s.

## Author Contributions

All authors listed have made a substantial, direct and intellectual contribution to the work, and approved it for publication.

## Conflict of Interest

The authors declare that the research was conducted in the absence of any commercial or financial relationships that could be construed as a potential conflict of interest.

## Footnotes

1 Here, guided by our abovementioned aim of generalizing L1 attrition to bilingualism research, we decided to adopt this rather broad definition as a starting point, but note that other definitions have been formulated. For an extensive review, see Köpke (2004).

2 With the exception. of a small set of attriting monolinguals who nonetheless, in the interpretation of the same experimenters reporting this evidence, are most probably experiencing indirect attrition via interaction with attriting bilinguals (Laufer and Baladzhaeva, 2015; Baladzhaeva and Laufer, 2018).
